# Adjuvant breast cancer chemotherapy during late-trimester pregnancy: not quite a standard of care

**DOI:** 10.1186/1471-2407-7-92

**Published:** 2007-05-30

**Authors:** Richard J Epstein

**Affiliations:** 1 Division of Haematology/Oncology, Department of Medicine, The University of Hong Kong, Pokfulam, Hong Kong

## Abstract

**Background:**

Diagnosis of breast cancer during pregnancy was formerly considered an indication for abortion. The pendulum has since swung to the other extreme, with most reviews now rejecting termination while endorsing immediate anthracycline-based therapy for any pregnant patient beyond the first trimester. To assess the evidence for this radical  change in thinking, a review of relevant studies in the fields of breast cancer chemotherapy, pregnancy, and drug safety was conducted.

**Discussion:**

Accumulating evidence for the short-term safety of anthracycline-based chemotherapy during late-trimester pregnancy represents a clear advance over the traditional norm of therapeutic abortion. Nonetheless, the emerging orthodoxy favoring routine chemotherapy during gestation should continue to be questioned on several grounds: (1) the assumed difference in maternal survival accruing from chemotherapy administered earlier – i.e., during pregnancy, rather than after delivery – has not been quantified; (2) the added survival benefit of adjuvant cytotoxic therapy prescribed within the hormone-rich milieu of pregnancy remains presumptive, particularly for ER-positive disease; (3) the maternal survival benefit associated with modified adjuvant regimens (e.g., weekly schedules, omission of taxanes, etc.) has not been proven equivalent to standard (e.g., post-delivery) regimens; and (4) the long-term transplacental and transgenerational hazards of late-trimester chemotherapy are unknown.

**Summary:**

Although an incrementally increased risk of cancer-specific mortality is impossible to exclude, mothers who place a high priority on the lifelong well-being of their progeny may be informed that deferring optimal chemotherapy until after delivery is still an option to consider, especially in ER-positive, node-negative and/or last-trimester disease.

## Background

Immediate chemotherapy may be clearly indicated for pregnant breast cancer patients with unresectable primary tumors or metastatic presentations, whereas operable tumors raise a more complex conflict of interest: the mother is threatened directly by cancer recurrence and hence, indirectly, by any treatment compromise made on behalf of the fetus. Decisions made in this context tend to be less drastic for the mother than for the fetus, given that the latter has more life-years to lose, and faces health hazards that are wholly avoidable. This risk imbalance is worsened by the weaker advocacy position of the fetus, who tends not to be considered the primary 'client' in such oncologic scenarios.

## Discussion

**Figure 1:**
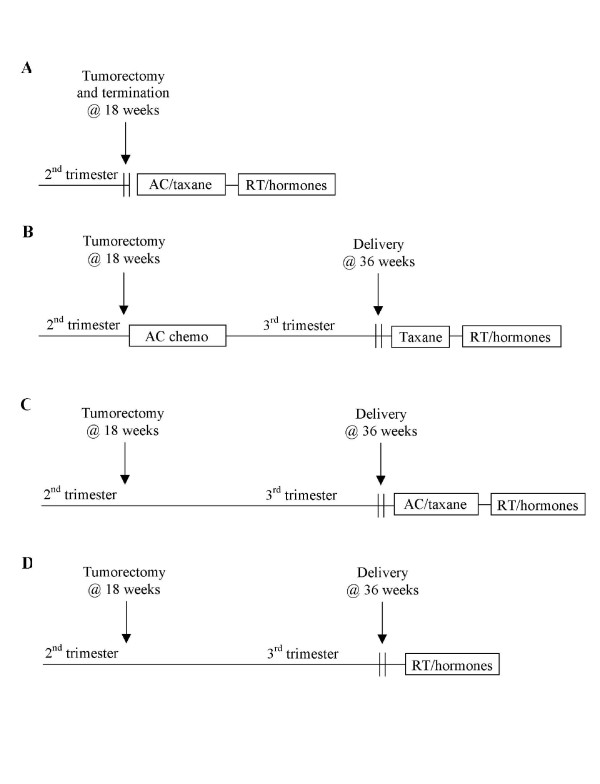
Schematic representation of patient choices for adjuvant therapies in resectable primary breast cancer diagnosed in the second or third trimesters. *A*, Immediate termination; *B*, Chemotherapy during pregnancy; *C*, Chemotherapy after pregnancy; *D*, No chemotherapy.

Recent studies have emphasized the safety of adjuvant anthracycline-based drug therapy during the second and third trimesters [1-5]. The post-resection dilemma in late-trimester cases can thus be reduced to three choices: termination of pregnancy followed by state-of-the-art adjuvant treatment; continuation of pregnancy with immediate administration of modified chemotherapy regimens that typically lack antimetabolites [6,7], taxanes [2] and trastuzumab [8] due to safety uncertainties; or continuation of pregnancy with optimal  chemotherapy given after delivery (Figure 1). Exclusive prioritization of decision-making towards the physical health of the mother favors termination followed by standard treatment, whereas prioritization towards the physical health of the fetus favors deferral of all nonsurgical treatments until after delivery. Many oncologists now see the option of second- and third-trimester cytotoxic therapy (hereinafter termed gestational chemotherapy) as a happy medium between these conservative and pro-active management positions, not least because of carefully-worded endorsements of anthracycline-based therapies as capable of being given with "minimal complications of labor and delivery" [1] or "minimal risk to the fetus" [2].

### Therapeutic delay

A central belief underlying the new enthusiasm for gestational chemotherapy is that delay of adjuvant cancer treatment is substantially deleterious to maternal outcomes. Taken to extremes, this assumption must be valid: if adjuvant treatment is delayed until metastases appear, the opportunity for cure has been lost. Such logic likewise supports the utility of therapeutic abortion which, uniquely, permits full deployment of all adjuvant modalities according to standard time-schedules. Relevant to this, however, no survival benefit attributable to therapeutic abortion has yet been proven [5],  despite the fact that this option enables the most standard adjuvant therapy  scheduling.

At present, there are no controlled data defining the extent to which a less extreme delay in the initiation of adjuvant therapy impairs overall survival outcomes. Retrospective studies have indicated that adjuvant chemotherapy delays of up to three months have no discernible effect on prognosis [9,10]; only in very advanced disease has delay in initiating adjuvant chemotherapy been associated with any impairment of disease-free survival [11], though other groups have failed to demonstrate impaired overall survival in any patient subset [12]. Using a model that quantifies risk increments in patients deferring breast cancer chemotherapy until after delivery, Nettleton *et al *estimated a modest 1.5–4.0% increased risk of transforming from axillary node-negative to -positive [13] in late-trimester patients, assuming a typical 10–12 week delay [14]. A large retrospective review of premenopausal patients indicated that only patients with ER-absent tumors are likely to benefit from early initiation of adjuvant chemotherapy [15].

### Therapeutic benefit

While it is reasonable to believe that gestational chemotherapy confers survival benefits similar to those apparent in overviews of nonpregnant patients receiving conventional chemotherapy [16], such overviews only calculate average effects, and should not be extrapolated to patient subsets affected by qualitative interactions [17]. Pregnancy represents exactly such an interaction: dramatic changes of mammary gland gene expression are induced by the presence of the fetus [18], reflecting corresponding changes in the tumor microenvironment. Breast cancer survival outcomes vary inversely with diagnostic proximity to earlier pregnancy [19,20], raising the possibility of biological resistance to adjuvant treatments. Since the benefit of chemotherapy tends to be lower for hormone-driven tumors in both the adjuvant [21] and neoadjuvant settings [22-24], and appears similar to that of ovarian suppression in premenopausal women with hormone-dependent disease [25,26], the question arises: for pregnant patients whose tumors express some hormone receptors, will adjuvant chemotherapy given "early" in the hormone-rich pregnancy environment necessarily be more effective than similar chemotherapy given after delivery; or could the opposite be the case?

A further worry raised by the vogue for gestational chemotherapy is the possibility that suboptimal regimens may be delivered, albeit with good intentions; in the worst of all possible worlds, such regimens could imperil the fetus without benefiting the mother. For example, Peccatori *et al *treated pregnant patients using weekly epirubicin 30–50 mg/m^2 ^with the aim of maximizing therapeutic effect while minimizing fetal drug exposure [27]. However, the adjuvant efficacy of such customized regimens – whether in pregnant or non-pregnant patients – is simply not known, though it is accepted that "low-dose" chemotherapy regimens are less effective. Other modifications of standard regimens – e.g., exclusion of antimetabolites and/or taxanes, lower dose-intensity and/or dose-density, or withholding of the humanized antibody trastuzumab – are popular in this setting, raising the question: is the administration of such 'modified' regimens during pregnancy aimed at treating the patient or the doctor? If a pregnant breast cancer patient does not receive chemotherapy of similar efficacy to that received by nonpregnant patients, should it be assumed that this deficiency will be rectified in the post-partum period – or would it be better to defer the entire treatment regimen until after delivery, at which time a more concentrated and intensive approach could plausibly improve survival benefits?

### Therapeutic safety

The usual endpoints that have been used to determine the fetal 'safety' of late-trimester chemotherapy include congenital malformations, developmental milestones and pediatric tumor rate. Indeed, the literature is replete with series [28,29] and case reports [30-32] of 'safe' treatment anecdotes where no obvious fetal malformation was documented following gestational chemotherapy. Yet as reassuring as these negative reports sound, they may not reflect the actual toxicity of late-trimester chemotherapy. Intrauterine growth retardation, miscarriages, stillbirths, pre-eclampsia and prematurity are increased in pregnancies involving cytotoxic treatment [33], yet are not ranked as malformations or teratogenicity. As listed in Table 1, more subtle long-term deficits in transplacentally-exposed progeny – such as reduced fertility, fine cognitive deficiencies, more frequent adult solid tumors, diminished longevity, or transgenerational carcinogenesis – may become apparent in "chemo babies" only after long-term surveillance [34].

A further safety-related concern is that a future pregnancy is possible, but not guaranteed. The summative risks of chemotherapy-induced infertility (say, 20%), early metastatic relapse (say, 20%) or marital problems (say, 10%) might be expected to reduce the certainty of having another baby, keeping in mind that the duration of any adjuvant hormonal therapy is likely to be five years or longer. Although it is difficult to quantify the relative net fertility outcomes of early vs. late chemotherapy in this context, it is certainly arguable that the fetus of a pregnant breast cancer patient may be, if anything, more precious to the mother than the average pregnancy, given the uncertain prognoses for both maternal survival and fecundity.

Moreover, patients may believe that the most painful choice – in this case, abortion, which is chosen in up to 50% of cases in many international series [35] – must be the best [36]. Clinicians aware of the anxieties facing the patient may feel obliged to offer active intervention ("at least we're doing something"), unintentionally biasing informed consent [37]. Patient acceptance of gestational chemotherapy may likewise have more to do with cultural norms of cooperation and punishment [38] than with logical consideration of all options. These concerns support reservations expressed by Barthelmes *et al *[39], Pautier *et al *[40] and Mathieu *et al *[41] about the trend towards routine immediate adjuvant chemotherapy for late-trimester breast cancer patients.

## Summary

Oncologists may be less familiar than obstetricians with the ethical complexities confronting pregnant patients. Management of cancer in pregnancy is complicated by the tendencies of clinicians to equate (maternal) risk with (maternal plus fetal) benefit, to assume that immediacy of action is always best, to hold the interests of the 'client' (mother) above those of any third party (fetus), and to imply that lack of proven danger equals safety. Basing standard recommendations on such convictions is tempting, but firmer knowledge of gestational breast cancer biology, measured therapeutic benefits, and longer-term toxicity data is needed before any such standard can be scientifically endorsed [42]. In the meantime, the debate will continue as to whether chemotherapy should be presumed 'innocent until proven guilty' or 'guilty until proven innocent': although the Hippocratic ethical maxim "First, do no harm" favors the latter from the viewpoint of the fetus, an assumption of gestational chemotherapy efficacy will favor the former from the viewpoint of the mother.

The inconvenient truth remains that gestational chemotherapy has not yet been proven to have net benefits (long-term maternal survival increments minus long-term fetal toxicities) exceeding those of delayed chemotherapy. Equally, however, there remains insufficient data to claim that delayed chemotherapy should be standard, and the issue is likely to remain unresolved. Nonetheless, deferral of chemotherapy to the post-partum period remains a valid and defensible option for oncologists to invite selected late-trimester breast cancer patients – e.g., those with hormone receptor-positive disease, node-negative staging, and/or no more than 12 weeks until delivery – to consider.

## Abbreviations

Nil

## Competing interests

The author(s) declare that they have no competing interests.

## Pre-publication history

The pre-publication history for this paper can be accessed here:



## Figures and Tables

**Table 1:** Expected *vs. *non-expected positive endpoints for gestational exposure to cytotoxic drugs.

**Expected effects of transplacental mutagens in late-trimester pregnancies**	
*Expected to be normal*	*Expected to be abnormal*

Congenital malformation rate	Abortion rate
Postnatal apgar indices	Prematurity, stillbirth
Neurologic milestones	Birth weight
Infant sexual/genital development	Adult fertility
Pediatric cancer rate	Adult cancer rate
Transgenerational malformation rate	Transgenerational cancer rate
Short-term cardiovascular function	Long-term cardiovascular function
Learning ability	Longevity
